# Expression of nectin-4 in papillary renal cell carcinoma

**DOI:** 10.1007/s12672-022-00558-2

**Published:** 2022-09-22

**Authors:** Stefanie Zschäbitz, Marie Mikuteit, Christine Stöhr, Edwin Herrmann, Iris Polifka, Abbas Agaimy, Lutz Trojan, Philipp Ströbel, Frank Becker, Christian Wülfing, Peter Barth, Michael Stöckle, Michael Staehler, Christian Stief, Axel Haferkamp, Markus Hohenfellner, Stefan Duensing, Stephan Macher-Göppinger, Bernd Wullich, Joachim Noldus, Walburgis Brenner, Frederik C. Roos, Bernhard Walter, Wolfgang Otto, Maximilian Burger, Andres Jan Schrader, Arndt Hartmann, Franziska Erlmeier, Sandra Steffens

**Affiliations:** 1 Dept. of Medical Oncology, National Center of Tumor Diseases, University Hospital Heidelberg, 69120 Heidelberg, Germany; 2 Department of Rheumatology and Immunology, Hanover Medical School, Carl-Neuberg-Straße 1, 30625 Hannover, Germany; 3 Dean’s Office – Curriculum Development, Hanover Medical School, 30625 Hannover, Germany; 4 Institute of Pathology, University Hospital Erlangen-Nuernberg, Friedrich Alexander University (FAU), 91054 Erlangen, Germany; 5 Department of Urology, University Hospital Muenster, 48149 Muenster, Germany; 6 Department of Urology, University Hospital Göttingen, 37075 Göttingen, Germany; 7 Department of Pathology, University Hospital Göttingen, 37075 Göttingen, Germany; 8 Department of Urology and Pediatric Urology, University of Saarland (UKS), 66421 Homburg, Germany; 9 Department of Urology, University of Marburg, 35037 Marburg, Germany; 10 Department of Urology, University Hospital Munich, 81337 Munich, Germany; 11 Department of Urology, University Hospital Mainz, 55131 Mainz, Germany; 12 Department of Urology, University Hospital Heidelberg, 69120 Heidelberg, Germany; 13 Institute of Pathology, University Hospital Mainz, 55131 Mainz, Germany; 14 Department of Urology and Pediatric Urology, University Hospital Erlangen-Nuernberg, Friedrich Alexander University Erlangen-Nürnberg, 91054 Erlangen, Germany; 15 Department of Urology, Marien Hospital Herne, Ruhr University Bochum, 44625 Herne, Germany; 16 Department of Urology, University Hospital Mainz, 55131 Mainz, Germany; 17 Department of Urology, University Hospital Frankfurt, 60590 Frankfurt, Germany; 18 Department of Urology, Caritas St. Josef and University, 93053 Regensburg, Germany; 19 Present Address: Institute of Urology, Prosper-Hospital GmbH, 45659 Recklinghausen, Germany; 20 Present Address: Urological Group and Clinic Derouet/Pönicke/Becker, Boxberg Centre, 66538 Neunkirchen, Germany; 21 Present Address: Department of Urology, Asklepios Clinics Altona, 22763 Hamburg, Germany; 22 Present Address: Institute of Pathology/Gerhard-Domagk Institute, University Hospital Muenster, 48149 Muenster, Germany; 23 Present Address: Department of Urology and Pediatric Urology, University Hospital Mainz, 55131 Mainz, Germany; 24 Present Address: Department of Gynecology, University of Mainz, 55131 Mainz, Germany; 25 Present Address: Department of Urology, Kreiskliniken Altötting-Burghausen, 84489 Burghausen, Germany; 26 Present Address: Department of Rheumatology and Immunology, Medical School Hannover, 30625 Hannover, Germany; 27 Present Address: Department of Rheumatology and Immunology, Hanover Medical School, 30625 Hannover, Germany

**Keywords:** Nectin 4, Papillary renal cell carcinoma

## Abstract

**Background:**

Nectin-4 contributes to tumor proliferation, lymphangiogenesis and angiogenesis in malignant tumors and is an emerging target in tumor therapy. In renal cell carcinoma (RCC) VEGF-directed tyrosine kinase inhibitors and checkpoint inhibitors are currently treatments of choice. Enfortumab vedotin-ejf (EV) is an antibody drug conjugate that targets Nectin-4. The aim of our study was to investigate the expression of Nectin-4 in a large cohort of papillary RCC specimens.

**Patients and methods:**

Specimens were derived from the PANZAR consortium (Erlangen, Heidelberg, Herne, Homburg, Mainz, Mannheim, Marburg, Muenster, LMU Munich, TU Munich, and Regensburg). Clinical data and tissue samples from n = 190 and n = 107 patients with type 1 and 2 pRCC, respectively, were available. Expression of Nectin-4 was determined by immunohistochemistry (IHC).

**Results:**

In total, Nectin-4 staining was moderately or strongly positive in of 92 (48.4%) of type 1 and 39 (36.4%) type 2 of pRCC cases. No associations between Nectin-4 expression and age at diagnosis, gender, grading, and TNM stage was found. 5 year overall survival rate was not statistically different in patients with Nectin-4 negative versus Nectin-4 positive tumors for the overall cohort and the pRCC type 2 subgroup, but higher in patient with Nectin-4 positive pRCC type 1 tumors compared to Nectin-4 negative tumors (81.3% vs. 67.8%, p = 0.042).

**Conclusion:**

Nectin-4 could not be confirmed as a prognostic marker in pRCC in general. Due to its high abundance on pRCC specimens Nectin-4 is an interesting target for therapeutical approaches e.g. with EV. Clinical trials are warranted to elucidate its role in the pRCC treatment landscape.

## Introduction

Renal cell carcinoma (RCC) divide into different subtypes [1]: Clear cell RCC (ccRCC) is the most common type, followed by papillary RCC (pRCC) with approximately 10–15% of all RCC cases [2, 3]. pRCC has been traditionally classified into two subtypes, type 1 and type 2. However, pRCC is a heterogeneous disease and subtyping a matter of debate. Historically, type 1 pRCC has been associated with more favorable clinical outcomes compared to type 2 pRCC [4–6]. Mutations in the MET (mesenchymal–epithelial transition) gene and gain of chromosome 7 are common in type 1 pRCC [7]. In type 2 pRCC, a broad variety of genetic alterations can be detected [8]. In localized disease tumor resection is the treatment of choice. In metastatic disease systemic therapy is the standard of care. Whereas in ccRCC first line treatment consists of combination treatment with either dual immunotherapy (nivolumab/ ipilimumab) [9] or tyrosine kinase inhibition plus either PD-1 or PD-L1 inhibition [10–12], for pRCC single agents are recommended at this time [13] and cabozantinib is the treatment of choice. Alternatives include sunitinib, savolitinib (however not yet approved in this indication) and pembrolizumab. Clinical trials e.g. SUNIFORECAST are ongoing and are investigating whether patients with pRCC might benefit as well from combination strategies [14].

The nectin family consists of four nectins (Nectin-1–4) and five nectin-like molecules (Necl1–5). These molecules are calcium-independent immunoglobuline-like transmembrane proteins that regulate intercellular junctions and tissue morphogenesis [15]. Nectin-4 is also known as Poliovirus receptor like 4 (PVRL4). In contrast to Nectins-1–3 that are physiologically expressed, Nectin-4—aside from embryogenesis—is almost exclusively present on malignant cells [16]. It contributes to tumor proliferation, lymphangiogenesis and angiogenesis in vitro and in vivo [17–19].

Nectin-4 has been identified as a prognostic marker in different cancers such as esophageal, lung, breast and pancreatic cancer [17, 18, 20–22].

Nectin-4 can be detected on the surface of almost all urothelial cancer (UC) cells and in most variant bladder cancer types [23]. Therefore, Nectin-4 has attracted attention as a therapeutical target and has entered clinic with the approval of enfortumab vedotin-ejf (EV). EV is an antibody drug conjugate that consists of an anti-nectin-4-antibody conjugated to the microtubule-disrupting agent monomethyl auristatin E. It has proven superior efficacy (overall survival (OS), progression free survival (PFS) and overall response rate (ORR)) in platinum- and immunotherapy refractory bladder cancer and is currently investigated in combination strategies and early treatment lines in UC [24]. In the pivotal dose escalation/ expansion phase I trial EV-101, 201 patients with Nectin-4 positive tumors were enrolled, among them 155 patients with heavily pretreated metastatic UC whose data were reported [25]. However, the efficacy of EV in Nectin-4 positive non-urothelial tumors is currently not known.

In this study, we investigate the expression of Nectin-4 in a large cohort of pRCC.

## Materials and methods

### Patient cohort and tumor characteristics

Specimens were obtained from the PANZAR consortium—a collaboration between the following institutions (in alphabetical order): Erlangen, Heidelberg, Herne, Homburg, Mainz, Mannheim, Marburg, Muenster, LMU Munich, TU Munich, and Regensburg [26–29]. The cohort consists of 368 patients. Tissue samples from a total of 297 patients were available for Nectin-4 staining and retrospective analysis of clinical data. The study was performed according to the standards of the Declaration of Helsinki and in concordance with each local ethics committee.

Kidney surgery was performed between 1994 and 2007. Representative material of the pRCC tumors was selected and tissue microarrays were constructed. For each patient’ sample, pathological TNM staging/ grading according to 2002 TNM classification and division into the papillary subtype according to 2004 WHO tumor classification were performed. All specimens were reviewed again in 2018 according to the at the time valid tumor classification by an experienced uropathologist (AH). Clinical and pathological characteristics of 297 patients are presented in Table 1.

**Table 1:** Characteristics of patients with papillary renal cell carcinoma type 1 and type 2

Variable	pRCC, all n = 297 (100%)	pRCC type 1 *n* = 190 (67.8%)	pRCC type 2 *n* = 107 (32.2%)	p
Age^a^, median (IQR), years	63.3 (55.0–71.0)	63.0 (54.2–70.0)	66.0 (57.0–73.1)	0.102^b^
Sex				0.741^c^
Female, *n* (%)	52 (17.5)	33 (17.4)	19 (17.8)	
Male, *n* (%)	190 (64.0)	127 (66.8)	63 (58.9)	
NE, *n* (%)	55 (18.5)	30 (15.8)	25 (23.4)	
T-stage				< 0.0001^d^
pT1, *n* (%)	142 (47.8)	106 (55.8)	36 (33.6)	
pT2, *n* (%)	51 (17.2)	37 (19.5)	14 (13.1)	
pT3, *n* (%)	46 (15.5)	17 (8.9)	29 (27.1)	
pT4, *n* (%)	1 (0.3)	0 (0.0)	1 (0.9)	
pTx, *n* (%)	57 (19.2)	30 (15.8)	27 (25.5)	
Grade				< 0.0001^d^
G1, *n* (%)	48 (16.2)	48 (25.3)	0 (0.0)	
G2, *n* (%)	125 (42.1)	106 (55.8)	19 (17.8)	
G3, *n* (%)	85 (28.6)	19 (10.0)	66 (61.7)	
Gx, *n* (%)	39 (13.1)	17 (8.9)	22 (20.6)	
LN metastasis^a^				< 0.0001^c^
N − , *n* (%)	274 (92.3)	184 (96.8)	90 (84.1)	
N+ , *n* (%)	23 (7.7)	6 (3.2)	17 (15.9)	
Distant metastasis^a^				< 0.0001^c^
M −, *n* (%)	218 (73.4)	155 (81.6)	63 (58.9)	
M+ , *n* (%)	16 (5.4)	2 (1.1)	14 (13.1)	
Mx, *n* (%)	63 (21.2)	33 (17.4)	30 (28.0)	
Locally or advanced				< 0.0001^c^
pT1/pT2 N0 M0, n (%)	187 (63.0)	140 (73.7)	47 (43.9)	
pT3/pT4 and/or N1 and/or M1, n (%)	48 (16.2)	18 (9.5)	30 (28.0)	
NE, n (%)	62 (20.9)	32 (16.8)	30 (28.0)	

*IQR* inter quartile range, *M−* no evidence of metastatic diseases, *M*+ evidence of metastatic disease, *N−*  lymph node status unknown or tumor cells absent from regional lymph nodes, *N*+ regional lymph node metastasis present, *NE* not evaluable, *pRCC*.papillary renal cell carcinoma

^a^at time of kidney surgery

^b^Mann-Whitney-U test

^c^Fisher exact test

^d^Chi square test

### Histopathology and immunohistochemistry (IHC)

Expression of Nectin-4 was determined by immunohistochemistry (IHC). Histopathology and IHC were performed as previously described [30–32]. Briefly, for IHC, 2 µm TMA slides have been stained for Nectin-4 (Anti-Nectin-4 antibody, abcam, ab192033, dilution 1:100). The antibody became implemented for 30 min after heat pretreatment at 120 °C for five minutes with Tris–EDTA buffer pH 9 and peroxidase blocking (Dako, Hamburg, Germany). Incubation with a horseradish peroxidase (HRP)-categorized secondary antibody polymer (EnVision, Dako) was performed for 30 min followed by adding a diaminobenzidine (DAB) substrate chromogen solution (Dako) for 10 min and counterstaining for 1 min with hematoxylin (Merck, Darmstadt, Germany). Incubation procedures were performed at room temperature. Positive controls in addition to negative control slides without the addition of primary antibody have been included for every staining experiment. Images were captured under a Leitz ARISTOPLAN light microscope (Leica Microsystems, Germany) with a×10 eyepiece, a 22-mm field of view and×40 objective lens (Plan FLUOTAR × 40/0.70). Tissue sections were analyzed in a blind way by a pathologist (FE).

The staining reaction was classified according to a semi-quantitative IHC reference scale as previously described [33, 34]. Nectin-4 was localized primarily in the cytoplasm and membrane.

The staining intensity was scored from 0 to 3 (0 = no staining, 1 = weak staining, 2 = moderate staining, 3 = strong staining). 87 samples showed no staining, 79 cases showed a weak staining, 78 cases showed a moderate staining, and 53 cases showed a strong staining intensity, respectively. The area of staining was evaluated in percent (0–100%), a staining intensity score was defined by multiplying the score with the stained area [35, 36]. Given the absence of normative data on cell membrane or cell cytoplasm staining intensity in the literature, values in our patient collective were dichotomized using the median of observed distribution as the cut off. A Nectin-4 staining lower or equal to the median was defined as Nectin-4 low, and a staining higher than the median was defined as Nectin-4 high.

### Statistical analysis

OS was censored in the absence of death at the last date of follow-up. Duration of follow-up was calculated from the date of surgery to either date of death or last known follow-up. Kaplan–Meier survival times were estimated, with subgroups being compared using the log-rank test. Chi-square, Fisher's exact tests, Mann–Whitney U-test, or independent t-test were used as appropriate, to compare between patient/ tumor characteristics and the corresponding subgroup with or without Nectin-4 expression. SPSS 27.0 (Armonk, NY, USA) was used for statistical assessment. Two-sided p-values below 0.05 were considered statistically significant.

## Results

### Expression of nectin-4 in papillary renal cell carcinoma

**Fig. 1:**
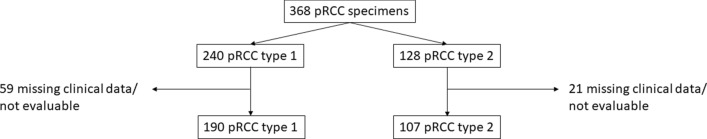
Consortium diagram of PANZAR cohort

Nectin-4 staining was evaluable in 190 of 240 patients with pRCC type 1 and in 107 of 128 patients with pRCC type 2 from the PANZAR cohort (Fig. 1).

**Fig. 2:**
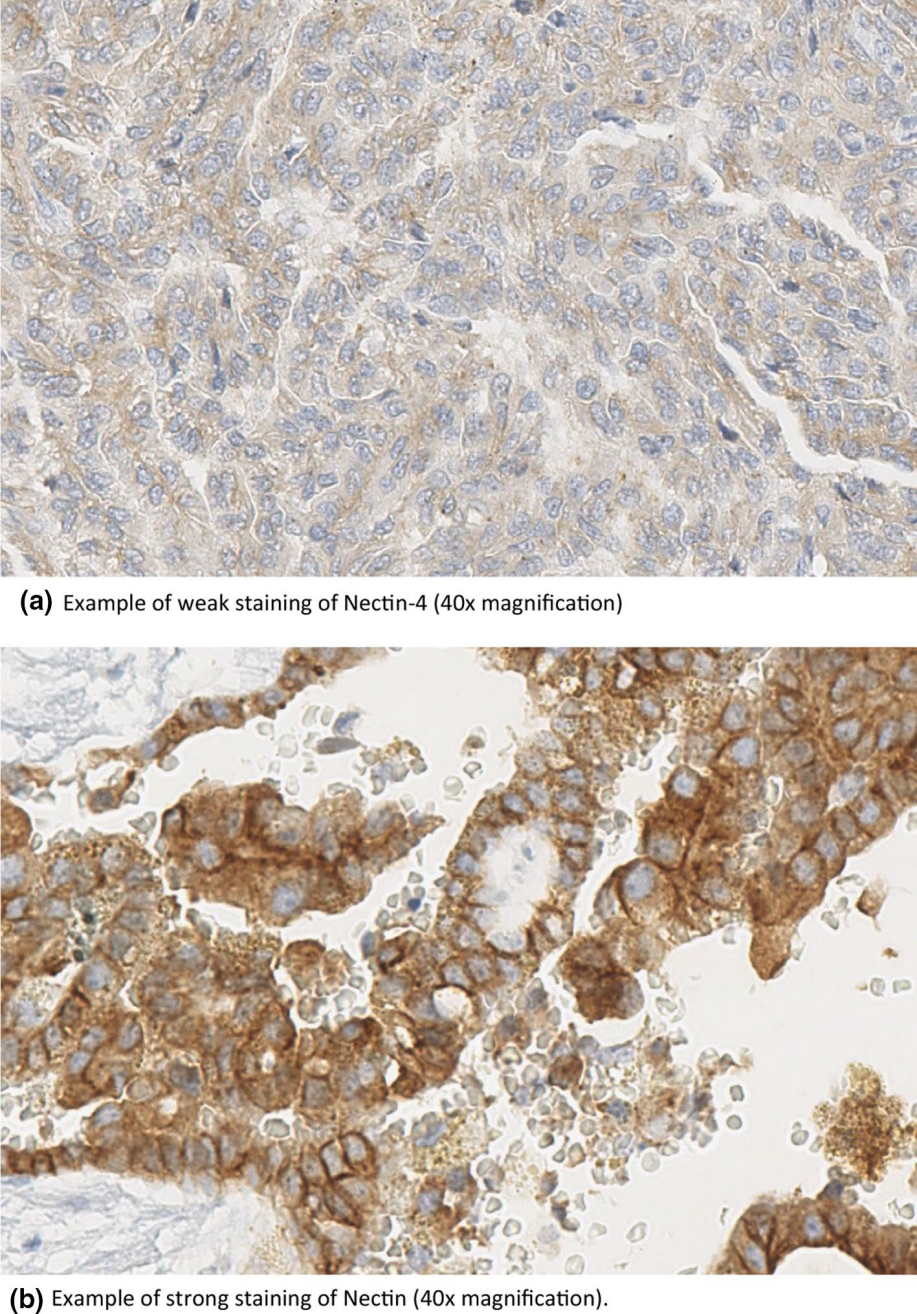
Immunohistochemical staining of Nectin-4 **a** Example of weak staining of Nectin-4 (40×magnification) **b** Example of strong staining of Nectin (40×magnification)

Clinico-pathological characteristics of the patients and their tumors are presented in Table 1. Staining for Nectin-4 was strongly or moderately positive in 131 (44.1%) individuals in the overall cohort (Fig. 2). We found no differences regarding age at diagnosis, gender, grade, T stage, N stage and M stage between Nectin-4 strong or moderate positive and low positive or negative pRCC tumors (Table 2). In total, Nectin-4 staining was strong or moderate positive in 92 (48.4%) of type 1 pRCC specimens. We did not detect an association between Nectin-4 expression and age at diagnosis, gender, grading, and TNM stage in the pRCC type 1 cohort (Table 3). Nectin-4 staining was strong or moderate positive in 39 (36.4%) of type 2 pRCC specimens. The subgroup analyses of the pRCC type 2 for Nectin expression showed no statistically significant difference in age, gender, grading, T stage, N stage nor M stage (Table 3).

**Table 2:** Characteristics of patients with papillary renal cell carcinoma in dependence of Nectin-4 expression

Variable	pRCC nectin-4 low positive or negative n = 166 (55.9%)	pRCC nectin-4 strong or moderate positive n = 131 (44.1%)	p-value
Age^a^, median (IQR) years	64.0 (54.8–71.3)	63.0 (55.5–70.0)	0.732^b^
Sex			0.638^c^
Female, *n* (%)	27 (16.3)	25 (19.1)	
Male, *n* (%)	107 (64.5)	83 (63.4)	
NE, *n* (%)	32 (19.3)	23 (17.6)	
T-stage			0.555^d^
pT1, *n* (%)	80 (48.2)	62 (47.3)	
pT2, *n* (%)	25 (15.1)	26 (19.8)	
pT3, *n* (%)	26 (15.7)	20 (15.3)	
pT4, *n* (%)	0 (0.0)	1 (0.8)	
pTx, *n* (%)	35 (21.1)	22 (16.8)	
Grade			0.533^d^
G1, *n* (%)	25 (15.1)	23 (17.6)	
G2, *n* (%)	64 (38.6)	61 (46.6)	
G3, *n* (%)	50 (30.1)	35 (26.7)	
Gx, *n* (%)	27 (16.3)	12 (9.2)	
LN metastasis ^a^			0.828^c^
N − , *n* (%)	154 (92.8)	120 (91.6)	
N+ , *n* (%)	12 (7.2)	11 (8.4)	
Distant metastasis ^a^			0.606^c^
M − , *n* (%)	116 (69.9)	102 (77.9)	
M+ , *n* (%)	10 (6.0)	6 (4.6)	
Mx*, n* (%)	40 (24.1)	23 (17.6)	
Locally or advanced			0.748^c^
pT1/pT2 N0 M0, n (%)	100 (60.2)	87 (66.4)	
pT3/pT4 and/or N1 and/or M1, n (%)	27 (16.3)	21 (16.0)	
NE, n (%)	39 (23.5)	23 (17.6)	

IQR inter quartile range, *M−  *no evidence of metastatic diseases, *M*+ evidence of metastatic disease, *N*−  lymph node status unknown or tumor cells absent from regional lymph nodes, *N*+  regional lymph node metastasis present, *NE* not evaluable, *pRCC* papillary renal cell carcinoma

^a^at time of kidney surgery

^b^Mann-Whitney-U test

^c^Fisher exact test

^d^Chi square test

**Table 3:** Characteristics of patients with papillary renal cell carcinoma type 1 and type 2 in dependence of Nectin-4 expression

Variable	pRCC type 1 Nectin-4 low positive or negative *n* = 98 (51.6%)	pRCC type 1 Nectin-4 strong or moderate positive *n* = 92 (48.4%)	*p*-value	pRCC type 2 Nectin-4 low positive or negative *n* = 68 (63.6%)	pRCC type 2 Nectin-4 strong or moderate positive *n* = 39 (36.4%)	*p*-value
Age^a^, median (IQR) years	63.7 (54.3–71.8)	62.0 (54.2–69.5)	0.499^b^	65.5 (56.1–71.3)	66.5 (59.1–75.6)	0.358^b^
Sex			1.0^c^			0.420^c^
Female, *n* (%)	16 (16.3)	17 (18.5)		11 (16.2)	8 (20.5)	
Male, *n* (%)	64 (65.3)	63 (68.5)		43 (63.2)	20 (51.3)	
NE, *n* (%)	18 (18.4)	12 (13.0)		14 (20.6)	11 (28.2)	
T-stage			0.470^d^			0.401^d^
pT1, *n* (%)	55 (56.1)	51 (55.4)		25 (36.8)	11 (28.2)	
pT2, *n* (%)	15 (15.3)	22 (23.9)		10 (14.7)	4 (10.3)	
pT3, *n* (%)	9 (9.2)	8 (8.7)		17 (25.0)	12 (30.8)	
pT4, *n* (%)	0 (0.0)	0 (0.0)		0 (0.0)	1 (2.6)	
pTx, *n* (%)	19 (19.4)	11 (12.0)		16 (23.5)	11 (28.2)	
Grade			0.420^d^	0.204^d^		
G1, *n* (%)	25 (25.5)	23(25.0)		0 (0)	0 (0)	
G2, *n* (%)	50 (51.0)	56 (60.9)		14 (20.6)	5 (12.8)	
G3, *n* (%)	12 (12.2)	7 (7.6)		38 (55.9)	28 (71.8)	
Gx, *n* (%)	11 (11.2)	6 (6.5)		16 (23.5)	6 (15.4)	
LN metastasis ^a^			0.683^c^			0.169^c^
N − , *n* (%)	94 (95.9)	90 (97.8)		60 (88.2)	30 (76.9)	
N+ , *n* (%)	4 (4.1)	2 (2.2)		8 (11.8)	9 (23.1)	
Distant metastasis ^a^			0.239^c^	0.760^c^		
M − , *n* (%)	75 (76.5)	80 (87.0)		41 (60.3)	22 (56.4)	
M+ , *n* (%)	2 (2.0)	0 (0.0)		8 (11.8)	6 (15.4)	
Mx*, n* (%)	21 (21.4)	12 (13.0)		19 (27.9)	11 (28.2)	
Locally advanced			0.620^c^			0.327^c^
pT1/pT2 N0 M0, n (%)	67 (68.4)	73 (79.3)		33 (48.5)	14 (35.9)	
pT3/pT4 and/or N1 and/or M1, n (%)	10 (10.2)	8 (8.7)		17 (25.0)	13 (33.3)	
NE, n(%)	21 (21.4)	11 (12.0)		18 (26.5)	12 (30.8)	

IQR inter quartile range, *M* no evidence of metastatic diseases, *M*+ evidence of metastatic disease, *N*−  lymph node status unknown or tumor cells absent from regional lymph nodes, *N*+ regional lymph node metastasis present, *NE* not evaluable, *pRCC* papillary renal cell carcinoma

^a^at time of kidney surgery

^b^Mann-Whitney-U test

^c^Fisher exact test

^d^Chi square test

### Expression of nectin-4 and clinical course

Median follow-up was 42.0 (IQR 21.0–81.3) months. At the time of last follow-up, 95 (57.2%) and 80 (61.1%) patients were alive, 33 (19.9%) and 22 (16.8%) patients had died and 38 (22.9%) and 29 (22.1%) patients were lost to follow up in the Nectin-4 low positive/ negative and Nectin-4 strong or moderate positive subgroups (p = 0.749, chi square).

**Fig. 3:**
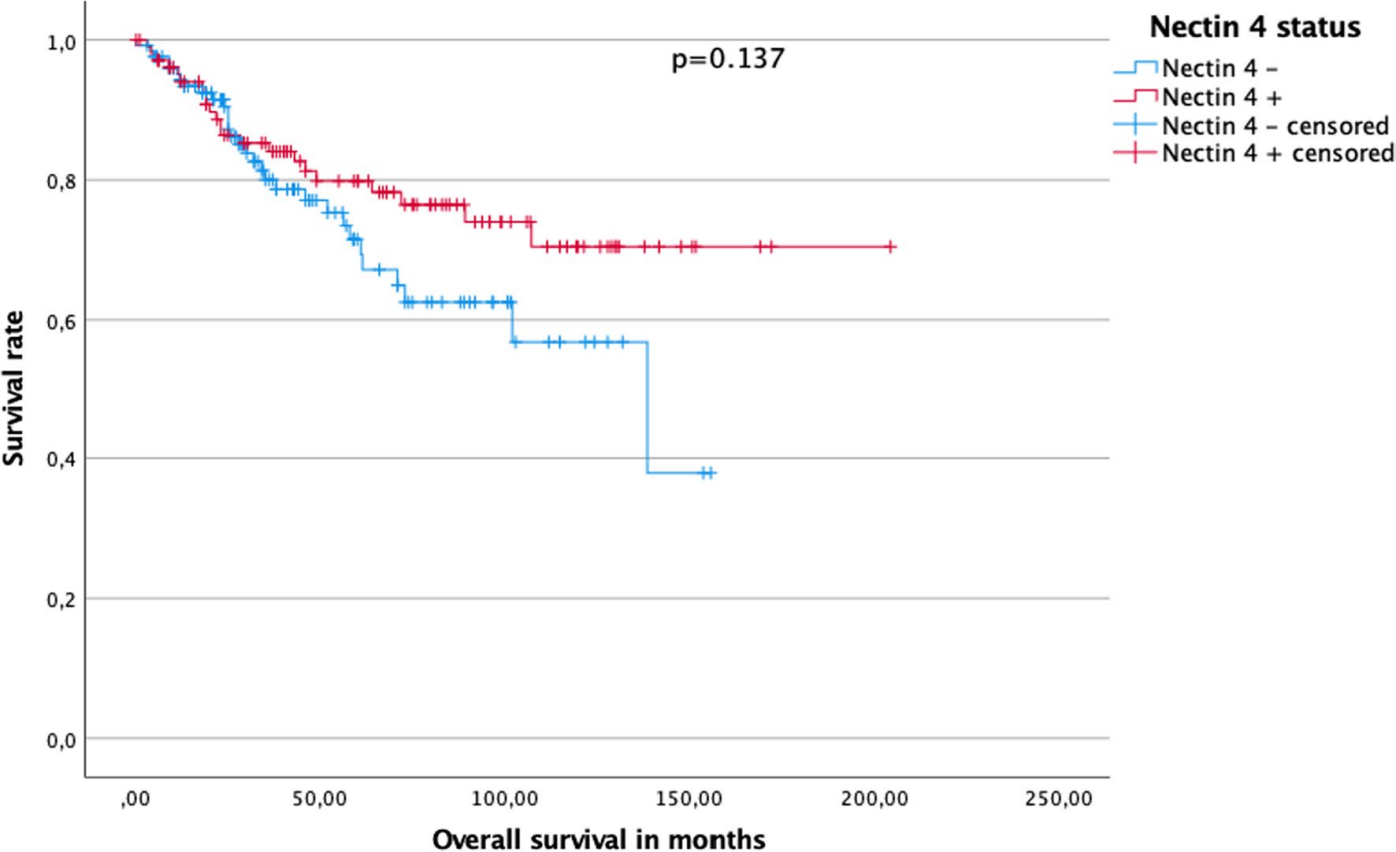
Cumulative survival in patients with papillary renal cell carcinoma depending on Nectin-4 status

Kaplan–Meier analysis disclosed a 5 year OS rate of 82.6% for patients with Nectin-4 low/positive negative tumors compared to 78.3% for patients with Nectin-4 strong or moderate positive tumors in pRCC (p = 0.185, log-rank). Median OS was 34.3 (IQR 20.9–62.6) months in the Nectin-4 low positive/negative group and 60 (IQR 23.0–97.5) months in the Nectin-4 strong or moderate positive group (see Fig. 3).

**Fig. 4:**
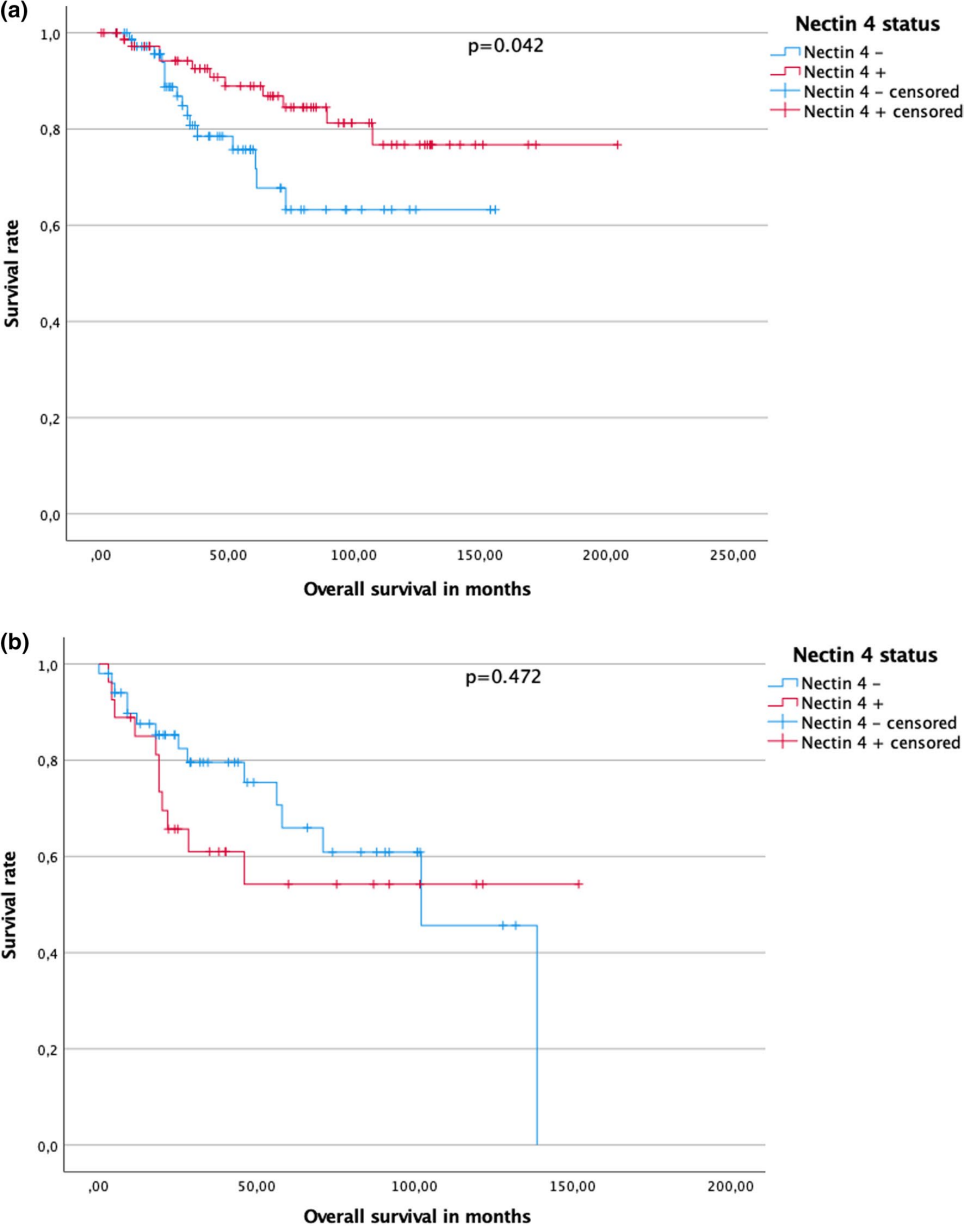
**a** Cumulative survival in patients with papillary renal cell carcinoma type 1 in dependence of Nectin-4 status. **b** Cumulative survival in patients with papillary renal cell carcinoma type 2 in dependence of Nectin-4 status

Median follow-up for patients with pRCC type 1 was 49.0 (IQR 25.0–84.5) months. At the time of last follow-up, 64 (69.6%) patients were alive, 11 (12.0%) patients had died, and 17 (18.5%) patients were lost to follow up in the Nectin-4 strong or moderate positive subgroup. At the time of last follow-up, 60 (61.2%) patients were alive, 16 (16.3%) patients had died, and 22 (22.4%) patients were lost to follow up in the Nectin-4 low positive/negative subgroup (Nectin-4 strong or moderate positive vs. low positive/negative p = 0.470, chi square). Kaplan–Meier analysis disclosed a 5 year OS rate of 67.8% for patients with Nectin-4 low positive/negative tumors compared to 81.3% for patients with Nectin-4 strong or moderate positive tumors in pRCC type 1 (p = 0.042, log rank). Median OS was 36.0 (IQR 23.4–61.0) months in the Nectin-4 low positive negative group and 68 (IQR 33.1–106.3) months in the Nectin-4 strong or moderate positive group (see Fig. 4a).

Median follow-up for patients with pRCC type 2 was 29.0 (IQR 18–71.8) months. At the time of last follow-up 16 (41.0%) patients were alive, 11 (28.2%) had died, and 12 (30.8%) patients were lost to follow up in the Nectin-4 strong or moderate positive subgroup whereas 35 (51.5%) patients were alive, 17 (25.0%) patients had died, and 16 (23.5%) patients were lost to follow up in the Nectin-4 low positive/negative subgroup (Nectin-4 strong or moderate positive vs. low positive/negative p = 0.560, chi square). Kaplan–Meier analysis disclosed a 5 year OS rate of 45.7% for patients with Nectin-4 low positive/negative tumors compared to 54.2% for patients with Nectin-4 strong or moderate positive tumors (p = 0.472, log rank) Median OS was 29.0 (IQR 16.0–71.0) months in the Nectin-4 low positive negative group and 28.3 (IQR 19.0–75.3) months in the Nectin-4 strong or moderate positive group (see Fig. 4b).

## Discussion

This is the first study exploring the expression and prognostic relevance of Nectin-4 in pRCC patients. pRCC is the second most common type of RCC and is a heterogenous subtype historically subdivided into two groups, type 1 and type 2. Type 1 pRCC is characterized by alterations in the MET gene and associated with a more benign clinical course compared to pRCC type 2 [8]. In pRCC type 2 several genetic alterations have been found, among them *CDKN2A* silencing, *SETD2* mutations, *TFE3* fusions, and *fumarate hydratase* (*FH*) gene mutations. However, due to poor interobserver reproducibility, overlapping features of both subtypes, and the lack of proven clinical significance the new WHO classification system will waive for a division into the two subtypes in the near future. Therefore, we evaluated the prognostic effect of Nectin-4 in the overall pRCC cohort as well as for pRCC type 1 and 2 subgroups.

Our study showed that Nectin-4 was strong or moderate positive in 131 (44.1%) individuals in the overall cohort, in 92 (48.4%) of type 1 pRCC specimens, and in 39 (36.4%) of type 2 pRCC specimens. No correlations could be found for either age at diagnosis, gender, grade or stage between Nectin-4 strong or moderate positive or low positive/ negative tumors. 5 year OS rate in Kaplan–Meier analysis was not statistically different in patients with Nectin-4 low positive/ negative versus Nectin-4 strong or moderate positive tumors for the overall cohort and the pRCC type 2 subgroup. Patients with pRCC type 1 showed a higher 5 year-OS probability compared to patients with pRCC type 2 as expected and those with Nectin-4 strong or moderate positive tumors a significantly higher 5 year OS probability with 81.3% compared to 67.8% for patients with Nectin-4 low positive/ negative pRCC type 1 tumors. In line with our results, in a cohort of 148 patients with triple negative breast cancer higher staining intensity of Nectin-4 in immunohistochemical analysis was associated with a significantly better survival, lower T stage and lower pN stage [37]. In contrast hereto, M-Rabet et al. investigated Nectin-4 expression on mRNA and protein level in 5673 invasive breast cancer specimens [18]. They found that high Nectin-4-mRNA expression was a negative prognostic marker for metastasis free survival and that mRNA expression was positively correlated with protein expression. In 94 samples of esophageal cancer, patients with high Nectin-4 expression showed decreased OS (HR = 1.747; 95% CI 1.003–3.044, p < 0.05) [21]. Nishiwada et al. explored Nectin-4 in 123 samples of pancreatic cancer [17]. They found a positive correlation between Nectin-4 and Ki67 proliferation index and siRNA knockdown of Nectin-4 inhibited proliferation of pancreatic cancer cells in vitro. They could also demonstrate a correlation between expression of Nectin-4 with vascular endothelial growth factor (VEGF). Patients with low Nectin-4 expression had significantly longer survival times (682 versus 426 days, p = 0.013). In 87 samples with hepatocellular carcinoma higher Nectin-4 expression was associated with shorter recurrence free survival (17.73 months versus 25.79 months, p = 0.006) and median OS (21.92 months versus 31.32 months, p = 0.005) [38]. In summary, the prognostic relevance of Nectin-4 remains contradictory in general and we conclude that Nectin-4 could not be confirmed as a prognostic marker in pRCC.

The high proportion of Nectin-4 positive pRCC tumors, however, raises the question whether the use of Nectin-4 directed therapies could add to the armamentarium of agents in this rare tumor entity. Data on efficacy and safety of EV in cancers other than UC are sparse. Data from non-UC patients from the dose escalation/expansion phase I trial EV-101 part A have not been published to date. An open-label phase 2 multicohort study (NCT04225117) is currently ongoing and enrolling into 6 tumor-specific cohorts, among those hormone receptor-positive/human epidermal growth factor receptor 2-negative (HR+ /HER2-) and triple-negative breast cancer, squamous non-small cell lung cancer, non-squamous non-small cell lung cancer, head and neck cancer, and gastric/esophageal adenocarcinoma and squamous cell carcinoma. Another monocentric phase 2 trial (NCT04754191) investigates EV in metastatic castration prostate cancer. To our knowledge, both trials recruit patients independent of Nectin-4 status. Within the StrataPATH^™^ non-randomized, open-label trial platform EV will be investigated in patients with advanced solid tumors (non-UC) harboring Nectin-4 over-expression (NCT05097599).

In addition, novel Nectin-4 directed agents are under investigation in patients with Nectin-4 positive tumors, among them second-generation bicycle peptides (Bicycle^®^) that bind to Nectin-4 and are covalently attached to MMAE (NCT04561362) or CD 137 (NCT05163041) as well as Nectin-4/FAP-targeted fourth-generation CAR-T cells (NCT03932565). However, few of these clinical trials are open to patients with rare tumor entities such as pRCC emphasizing the importance of thorough characterization of such tumor types and implementation of basket trial designs that also allow recruitment of patients with rare cancer types.

Our study has several limitations: First, although this is the largest cohort of pRCC patients reported to date, case numbers are limited due to the low incidence of this tumor entity. Second, the methodology of IHC and the interpretation system are accompanied by a certain risk of error susceptibility. Third, representative tissue samples were used for TMA construction. However, it cannot be ruled out with certainty that Nectin-4 expression is inhomogeneous. Fourth, data was analyzed retrospectively.

In summary, we could not confirm Nectin-4 expression as a prognostic marker in pRCC in general. However, Nectin-4 is an interesting target for therapeutical approaches as it is almost exclusively expressed on malignant cells and shows a high abundance on pRCC specimens. Further studies are warranted to elucidate whether Nectin-4-directed drugs might represent a new class of drugs in the treatment of pRCC.

## Data Availability

The datasets generated during and/or analysed during the current study are available from the corresponding author on reasonable request.

## Abbreviations

ccRCC: Clear cell renal cell carcinoma
EV: Enfortumab vedotin-ejf
IHC: Immuno histochemistry
IQR: Inter quartile range
LN: Lymph node
MET: Mesenchymal–epithelial transition
NE: Not evaluable
Non-ccRCC: Non-clear cell renal cell carcinoma
ORR: Overall response rate
OS: Overall survival
PFS: Progression free survival
pRCC: Papillary renal cell carcinoma
PVRL4: Poliovirus receptor like 4
RCC: Renal cell carcinoma
SD: Standard deviation
TMA: Tissue microarray
TNM: Tumor/node/metastasis
UC: Urothelial cancer
VEGF: Vascular endothelial growth factor
WHO: World Health Organization
